# Bibliometric and visual analysis of nephrotoxicity research worldwide

**DOI:** 10.3389/fphar.2022.940791

**Published:** 2022-09-14

**Authors:** Tianmu He, Jingwen Ao, Cancan Duan, Rong Yan, Xiaomei Li, Liu Liu, Jianyong Zhang, Xiaofei Li

**Affiliations:** ^1^ School of Basic Medical Sciences, Guizhou Medical University, Guiyang, China; ^2^ School of Basic Medical Sciences, Zunyi Medical University, Zunyi, China; ^3^ School of Pharmacy and Key Laboratory of Basic Pharmacology Ministry Education and Joint International Research Laboratory of Ethnomedicine Ministry of Education, Zunyi Medical University, Zunyi, China; ^4^ Affiliated Hospital of Zunyi Medical University, Zunyi, China

**Keywords:** acute kidney injury, bibliometrics, nephrotoxicity mechanism, renoprotective strategies, hotspot

## Abstract

**Background:** Nephrotoxicity of drugs contributes to acute kidney injury with high mortality and morbidity, which crucially limits the application and development of drugs. Although many publications on nephrotoxicity have been conducted globally, there needs to be a scientometric study to systematically analyze the intellectual landscape and frontiers research trends in the future.

**Methods:** Publications on nephrotoxicity from 2011 to 2021 were collected to perform bibliometric visualization using VOSviewer, CiteSpace, and Scimago Graphica software based on the Web of Science Core Collection.

**Results:** A total of 9,342 documents were analyzed, which were primarily published in the United States (1,861), China (1,724), and Egypt (701). For institutions, King Saud University (166) had the most publications; Food and Chemical Toxicology, PLOS One, and Antimicrobial Agents and Chemotherapy were productive journals, primarily concentrating on the mechanisms of nephrotoxicity and renoprotective in cisplatin and antibiotics, especially in oxidative stress. Burst detection suggested that cisplatin, piperacillin-tazobactam, vancomycin-induced nephrotoxicity, antioxidants, and new biomaterials are frontiers of research.

**Conclusion:** This study first provides an updated perspective on nephrotoxicity and renoprotective strategies and mechanisms. This perspective may benefit researchers in choosing suitable journals and collaborators and assisting them in the deep understanding of the nephrotoxicity and renoprotective hotspots and frontiers.

## Introduction

The kidney, as the main excretory organ in the body, is more susceptible to chemical and drug toxicity (Nishinakamura, 2019). Valuable evidence demonstrate that nephrotoxicity is one of the common adverse effects of drugs and a major obstacle to drug development. Acute and chronic kidney injuries are mainly the result of nephrotoxicity, which affects approximately 13.3 million people around the world each year (Kellum et al., 2021; Pickkers et al., 2021), a significant statistic given that 75% of hospitalized patients may suffer nephrotoxicity exposure (Moffett and Goldstein, 2011; Davis-Ajami et al., 2016), which contributes to increasing a patient’s odds of developing acute kidney injury (AKI) by 53% (Ostermann et al., 2018). Importantly, the general population is often exposed to the nephrotoxicity of prescribed and over-the-counter drugs, natural products, supplements, herbal medicines, and imaging agents (Izzedine et al., 2009; Perazella and Reilly, 2011; Yang et al., 2018). For example, cisplatin can induce nephrotoxicity in 30–40% of patients, commonly causing AKI and renal failure (Volarevic et al., 2019). However, nephrotoxicity is a sophisticated toxic reaction, which involves many risks and complex processes, including congenital nephrotoxicity, patient-specific risk factors, metabolism, and excretion of drugs which may be potential causative agents of kidney damage (Perazella, 2018).

Modern toxicology evidence suggest that medications could induce kidney injury through continuous drug accumulation, drug-related immune effects, and drug-metabolite insolubility in the urine (Perazella, 2010; Perazella and Izzedine, 2015). The molecular mechanism of nephrotoxicity is commonly related to oxidative stress (OS), inflammation, apoptosis, and autophagy (Gao et al., 2021). Notably, the latest nephrotoxicity studies have commonly focused on the renoprotective mechanism, early warning, and nanomedicine of nephrotoxicity. Beclin-1, MiR-155, farnesoid X receptor, and mitochondrial homeostasis proteins may be novel therapy target strategies of nephrotoxicity through autophagy blockade, ferroptosis, and OS (Kim et al., 2022; Shi et al., 2022; Yin et al., 2022; Zhang et al., 2022). In addition, phosphatidylcholines and taurine may be early biomarkers of nephrotoxicity using metabolomics and mass spectrometry imaging approaches (Chen et al., 2022; Locci et al., 2022), while nuclear factor erythroid 2-related factor 2 (Nrf2) could predict the severity of nephrotoxicity using deep learning approach (Feng et al., 2022). Moreover, compound nanocapsules, selenium nanoparticles, and renalase agonist nanoparticles could effectively reduce nephrotoxicity by inhibiting cell death and OS (Alotaibi et al., 2022; Guo et al., 2022; Mehanna et al., 2022).

Although a large number of nephrotoxicity studies have been conducted currently, there has been no global study to examine and predict the frontiers of nephrotoxicity research. Therefore, a bibliometric analysis was performed for scientists to systematically review the nephrotoxicity research studies. Bibliometric analysis (Chen, 2004) is used to track developments and explore particular areas of knowledge in medicine fields (Lu et al., 2020; Dong et al., 2021; Huang et al., 2021; Zhu et al., 2021) using CiteSpace (Chen, 2006) and VOSviewer (Waltman, 2007) software. Notably, we intended to visually analyze nephrotoxicity research hotspots through the bibliometric approach to make suggestions and further perspective.

## Methods

**FIGURE 1:**
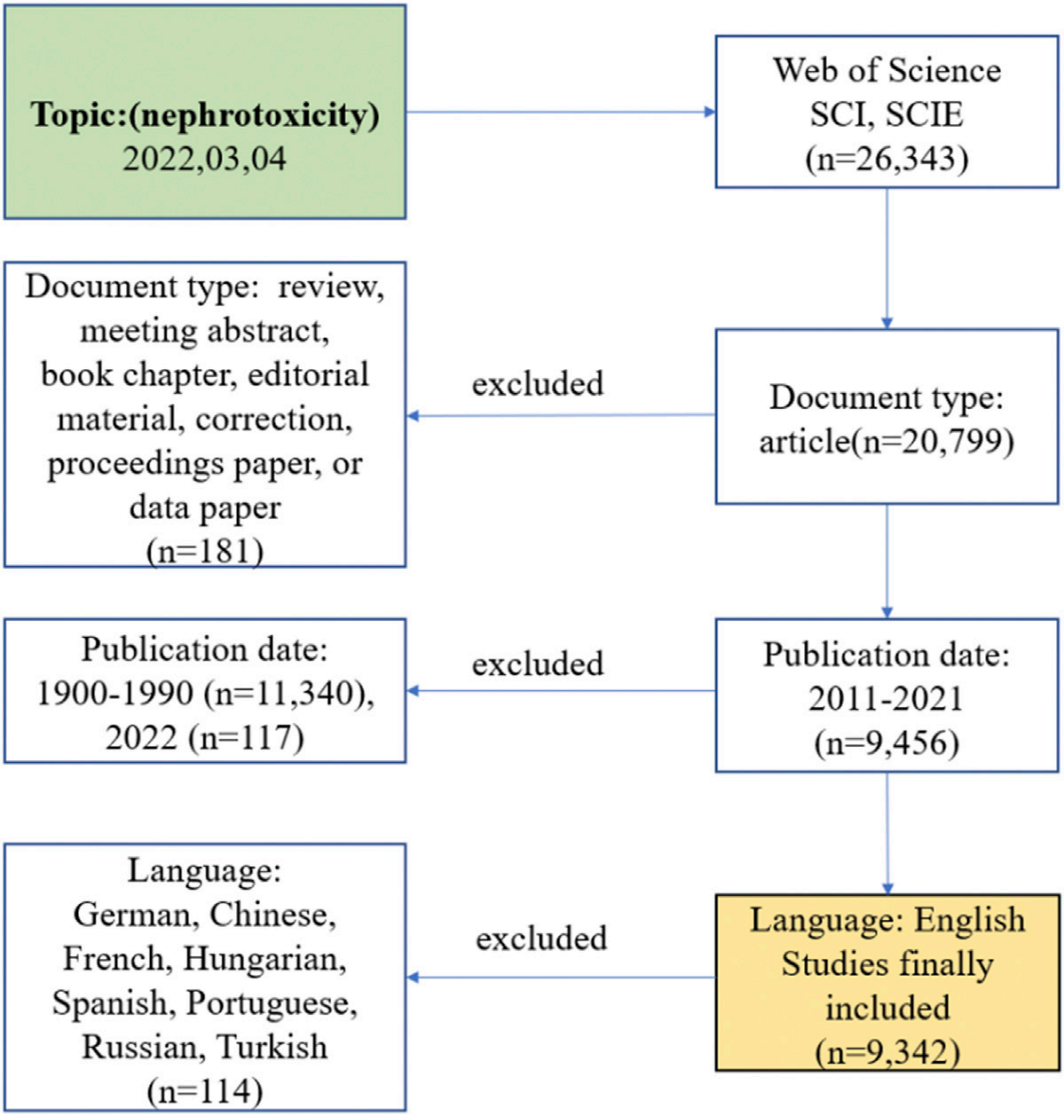
Frame flow diagram of nephrotoxicity search strategy from 2011 to 2021 based on Web of Science.

All the data were collected from the SCI-E and SSCI of the Web of Science Core Collection (WoSCC). The final suitable result was chosen by performing different search strategies, including various publication time periods or topics, and any divergences were settled by consultation with external specialists to reach consensus. Then, the retrieval strategy was stated as (topic = nephrotoxicity), (type = article), (year published = 2011–2021), and (language = English). The date of retrieval was 4 March 2022 by two researchers (Figure 1).

VOSviewer 1.6.16 and CiteSpace 5.8.R3 were applied to identify co-cited articles, keywords, countries, institutions, journals, authors, and reference bursts. The H-index, impact factor (IF), and category quartiles were collected from the Journal Citation Report (2020). The data of publications, citations, and polynomial trend lines were analyzed by Excel software. CiteSpace software was used to construct the hotspots and knowledge base map of nephrotoxicity research, and detect the centrality. VOSviewer was utilized to analyze the authorship and co-occurrence of keywords and visualize the frontiers of the nephrotoxicity field by clustering with different colors. Scimago Graphica 1.0.18 was used to visualize the collaborative relationships in countries/regions.

## Results

### Literature development trends

**FIGURE 2:**
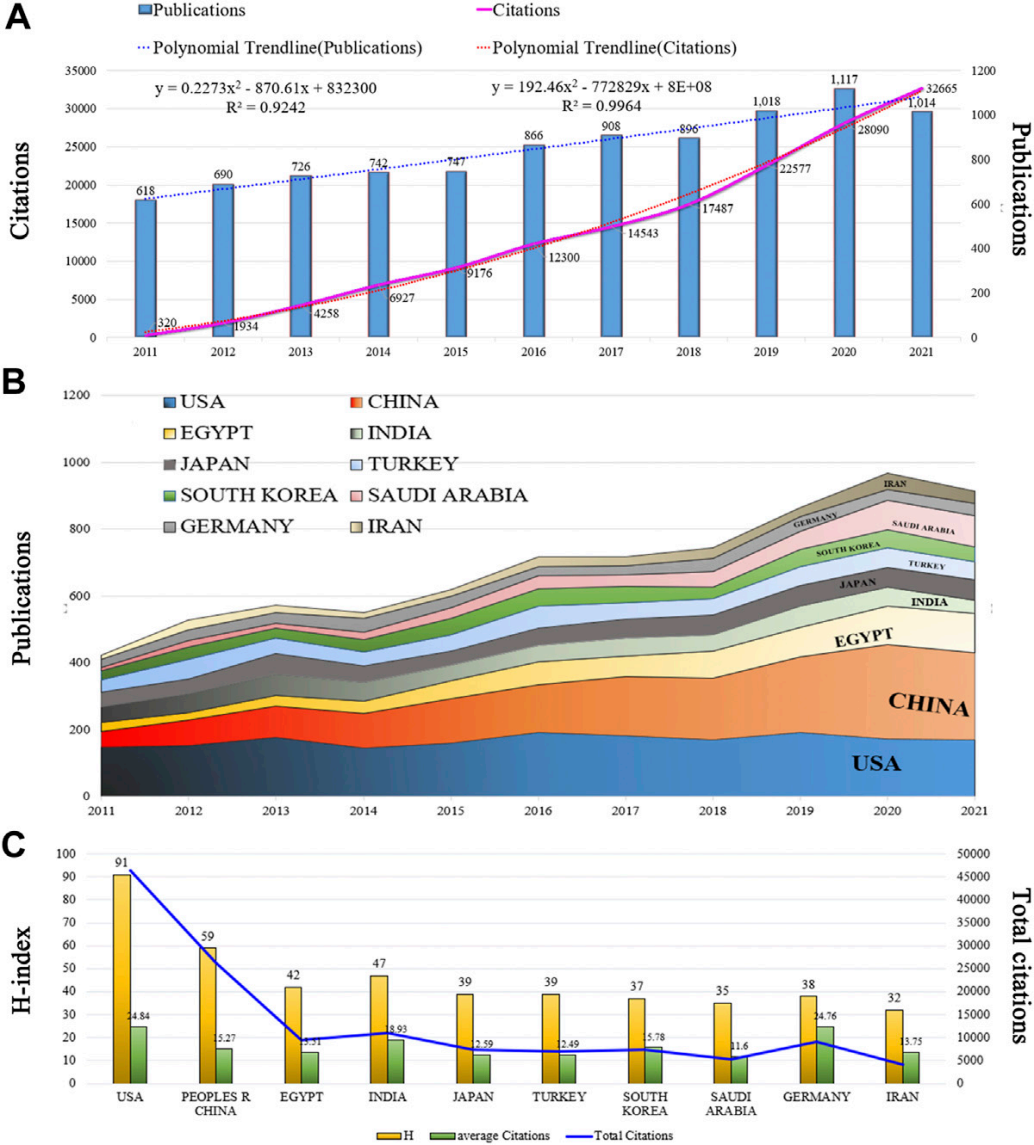
Trends in publications and citations of nephrotoxicity research. **(A)** Annual trends of global publications and citations. **(B)** Temporal trends of publications from the top 10 countries. **(C)** H-index, average citations (citations per article), and total citations of the top 10 countries.

A total of 9,342 articles on nephrotoxicity were published in 2011–2021 based on WoSCC, and the H-index count was 112. There were 91,626 and 16.46 total and mean citations, respectively, exhibiting an overall upward trend in the past decade (Figure 2A). From 2011 onward, the number of articles on nephrotoxicity increased with a continuous growth till 2021, and the publications in 2021 were nearly double that in 2011. In the past 5 years, research activity on nephrotoxicity peaked in terms of volume, with 4,953 articles being published. Furthermore, the annual number of citations also sharply increased from 2015 onward, with even more than 100 times the number of citations in 2021 than in 2011. The linear fitting of articles in nephrotoxicity showed a significant correlation (*R* ^2^ = 0.9964) between the year and the citations, attracting widespread attention from scientists in the nephrotoxicity field around the world.

### Geographic distribution

**FIGURE 3:**
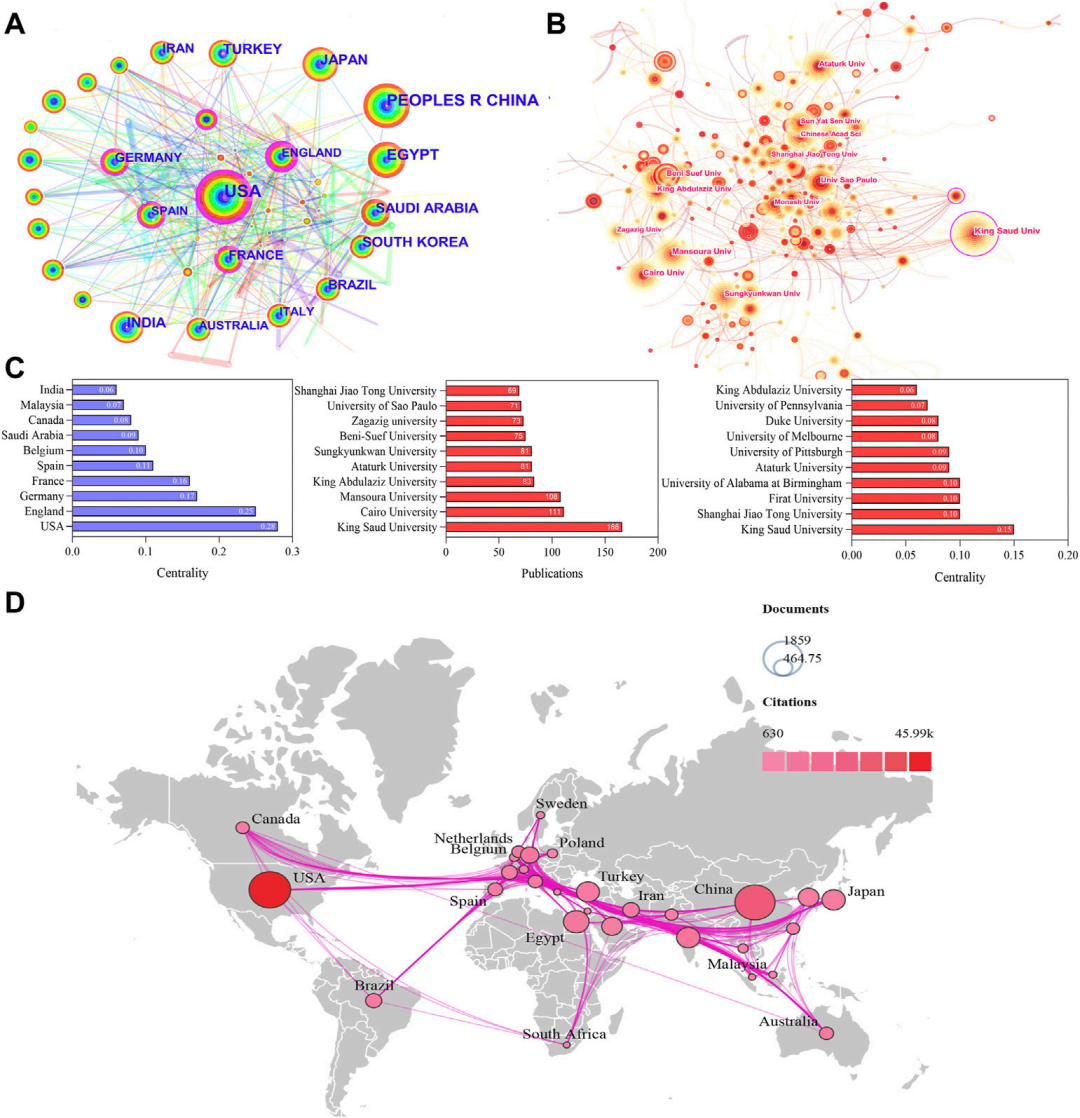
Visualization map of countries/regions and institutions involved in nephrotoxicity research. **(A)** Collaboration network of countries of CiteSpace. *N* = 123, *E* = 552. **(B)** Collaboration network of institutions of CiteSpace. *N* = 520, *E* = 978. (*N* represents the number of network nodes and *E* represents the number of connections). **(C)** Publications and centrality of countries/regions and institutions in nephrotoxicity research. **(D)** Collaborative relationships in countries/regions.

All literature was distributed among 123 countries/regions and 6,843 institutions. The top five countries of publications were the United States (1,861, 19.92%), China (1,724, 18.45%), Egypt (701, 7.50%), India (585, 6.26%), and Japan (583.6.24%); in the top 10 countries, 60% of the countries originated from Asia (Table 1). In addition, further effort was made to build the annual national publications (Figure 2B) and citations (Figure 2C) of the countries. The United States has published nearly 20% of all studies with a stable growth of publications in the last decade, but China has aroused the concern for nephrotoxicity research with a sharp increase of publications in nearly 5 years. The top three countries of mean citations were the United States (24.84), Germany (24.76), and India (18.93). Also, the top three countries of the H-index were the United States (H = 91), China (H = 59), and Egypt (H = 42). Among them, the United States, England, Germany, France, Spain, and Belgium were core nodes (centrality>0.1) marked with a purple circle (Figure 3A). The earlier mentioned results demonstrated that nephrotoxicity had received widespread attention from global scholars, and the United States and China were the leading contributors; furthermore, the United States represented a closer cooperation with each other in nephrotoxicity research (Figure 3D).

**TABLE 1:** Top 10 countries by publications, H-index, and citations in nephrotoxicity research.

Rank	Countries/regions	Publications	percentage of 9,342	H-index	Total citations	Average citations
1st	United States	1,861	19.92	91	46,743	24.93
2nd	China	1,724	18.45	59	26,665	15.35
3rd	Egypt	701	7.50	43	9,586	13.56
4th	India	585	6.26	47	11,162	18.98
5th	Japan	583	6.24	39	7,449	12.76
6th	Turkey	567	6.07	38	7,140	12.57
7th	South Korea	464	4.97	38	7,384	15.88
8th	Saudi Arabia	453	4.85	35	5,313	11.68
9th	Germany	365	3.91	48	9,171	25.20
10th	Iran	297	3.18	32	4,143	13.90

The top five publications of institutions were King Saud University (166), Cairo University (111), Mansoura University (108), King Abdulaziz University (83), and Ataturk University (81), and most of them originated from Egypt and Saudi Arabia (Figure 3C). In addition, King Saud University (0.15), Shanghai Jiao Tong University (0.10), Firat University (0.10), and University of Alabama at Birmingham (0.10) were the core nodes with a high centrality (Figure 3B), and most of them originated from the United States. The aforementioned results suggested that the institutions of Egypt and the United States were the main research forces. However, the centrality values were still low in general; thus, global cooperation should be strengthened in nephrotoxicity research field.

### Contributions of authors and co-cited authors

**FIGURE 4:**
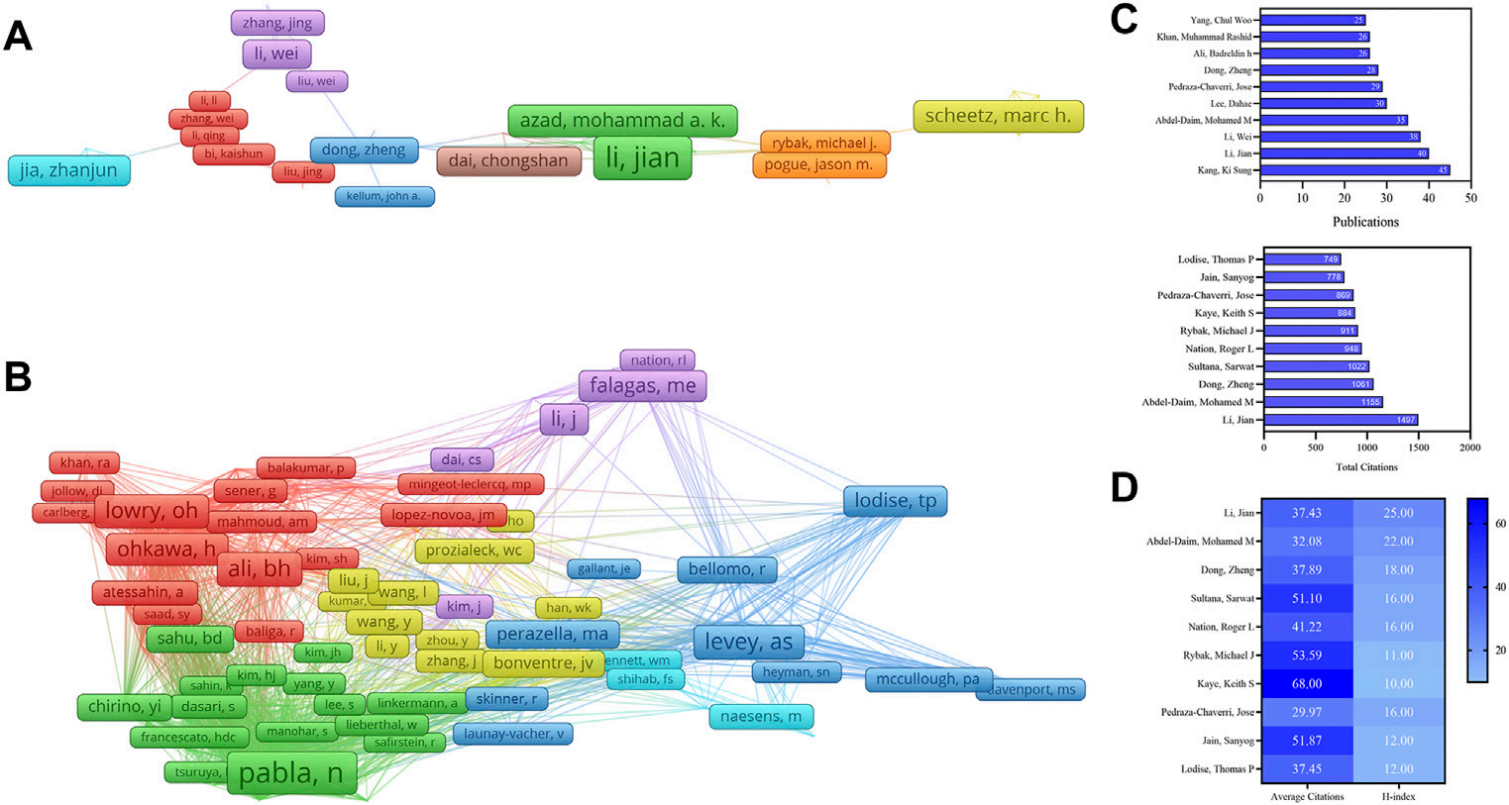
VOSviewer visualization map of authors and co-cited authors devoted to nephrotoxicity research. **(A)** Cooperation network of authors. Of the 47,803 authors, 144 had published at least 10 documents. **(B)** Co-citation network of authors. Of the 136,917 co-cited authors, 195 had at least 100 citations. Publications, total citations **(C)**, average citations, and H-index **(D)** of authors in nephrotoxicity research.

A total of 47,803 authors were obtained for the nephrotoxicity research. Kang, Ki Sung (45), the most productive author from South Korea, followed by Li, Jian (40), Li, Wei (38), Abdel-Daim, Mohamed M (35), and Lee, Dahae (30) (Table 2). In the total citation, the top three most cited authors were Li, Jian (1,497), Abdel-Daim, Mohamed M (1,155), and Dong, Zheng (1,061). Furthermore, Li, Jian’s H-index (25) was the highest (Figures 4C,D), who cooperated closely with Azad, Mohammad A. K and collaborated frequently with Li, Wei and Dong, Zheng (Figure 4A). In the co-citation network (Figure 4B), Pabla, Navjotsingh (831) had the highest co-citations, followed by Ramesh, Ganesan (600), and Ali, Badreldin H (575).

**TABLE 2:** Top 10 authors distributed by citations in nephrotoxicity research.

Rank	Cited author	Total citations	Average citations	H-index	Country/region
1st	Li, Jian	1,497	37.43	25	Australia
2nd	Abdel-Daim, Mohamed M	1,155	32.08	22	Egypt
3rd	Dong, Zheng	1,061	37.89	18	China
4th	Sultana, Sarwat	1,022	51.10	16	India
5th	Nation, Roger L	948	41.22	16	Australia
6th	Rybak, Michael J	911	53.59	11	United States
7th	Kaye, Keith S	884	68.00	10	United States
8th	Pedraza-Chaverri, Jose	869	29.97	16	Mexico
9th	Jain, Sanyog	778	51.87	12	India
10th	Lodise, Thomas P	749	37.45	12	United States

### Journal analysis

**FIGURE 5:**
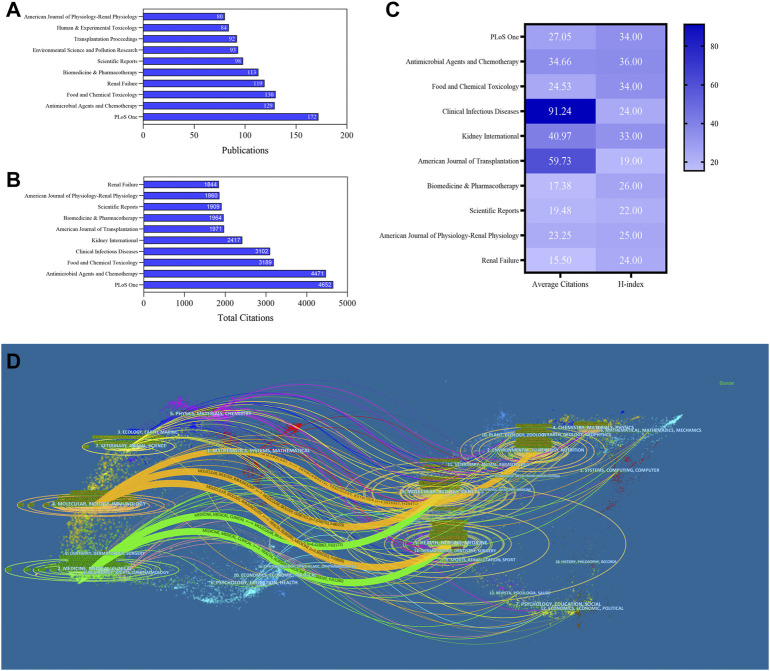
Publications **(A)**, total citations **(B)**, average citations, and H-index **(C)** of journals in nephrotoxicity research. **(D)** Dual-map overlay of journals in nephrotoxicity research. Left circles were targeted literature, while right circles were source literature.

A total of 1,756 journals were obtained, including 18 journals with more than 60 articles. The top three prolific and most citations journals were *PLOS One* (IF3.24), *Antimicrobial Agents and Chemotherapy* (IF5.191), and *Food and Chemical Toxicology*​ (IF6.023) (Figures 5A,B). Also, *Clinical infectious diseases* (IF9.079) had the highest average citations (91.24); most journals were classified in Q1 or Q2, suggesting that they were highly regarded for their research on nephrotoxicity.

The dual-map overlays showed the effective collaboration between publications and citing references in diverse fields (Figure 5D). Citing articles primarily centered on three fields: molecular, biology, and immunology; medicine, medical, and clinical; and veterinary, animal, and science. Among these fields, the circles representing the fields of medicine, medical, and clinical were larger, indicating that the numbers of coauthors and the numbers of published publications were relatively large. Also, the circle of health, nursing, and medicine was cited by publications in other fields, which played a crucial role in the cited references of nephrotoxicity research.

### Cluster analysis of co-occurrence keyword

**FIGURE 6:**
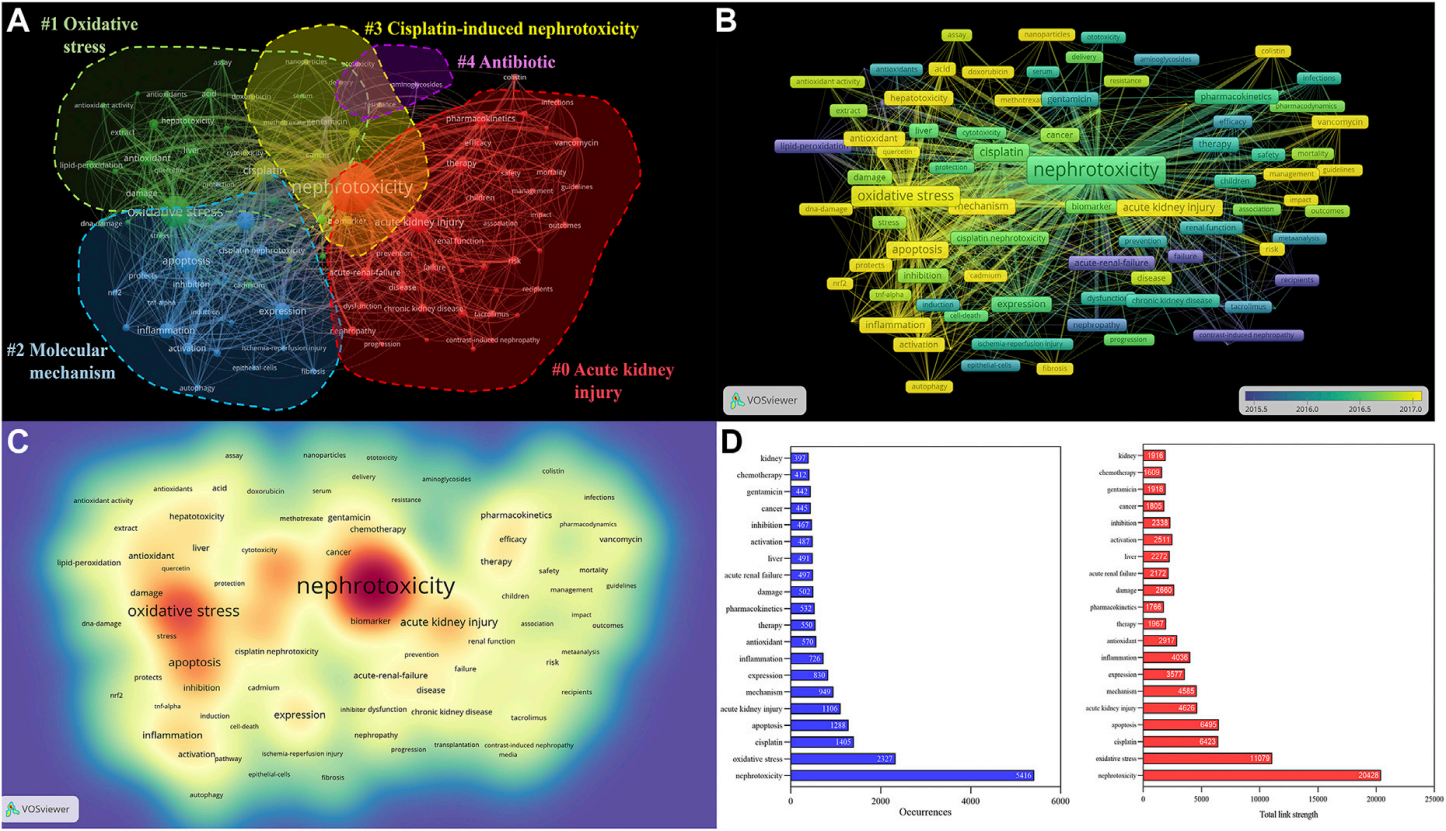
Analysis of all keywords in studies related to nephrotoxicity research. **(A)** VOSviewer visualization map of co-occurring keywords. Of the 23,900 keywords, 120 had at least 100 co-occurrences. **(B)** Overlay map of keywords. The closer to blue the keyword node color, the earlier the time of its occurrence. **(C)** Density map of keywords. **(D)** Occurrences and total link strength of keywords.

A map was then created by VOSviewer with 120 terms (23,900 in total), with at least 100 appearances per term (Figure 6D). Terms with comparable studies were merged under the same catalog, with four major clusters of “#0 acute kidney injury,” “#1 oxidative stress,” “#2 molecular mechanism,” “#3 cisplatin-induced nephrotoxicity,” and “#4 antibiotic” (Figure 6A). The major red cluster #0 was constituted by 45 items, including “acute renal injury,” “acute renal failure,” and “pharmacokinetics,” which mainly focused on the clinical therapy of nephrotoxicity. The green #1 and blue green #2 clusters primarily probed various toxic mechanisms of nephrotoxicity, including OS, inflammation, apoptosis, and autophagy. The time overlay map indicated that the signaling pathways and AKI still dominated in recent nephrotoxicity research (Figure 6B). Also, the frequency of the keywords was constructed to ascertain their density, and OS and AKI occupied the core part (Figure 6C).

### Analysis of highly cited and co-cited articles

**FIGURE 7:**
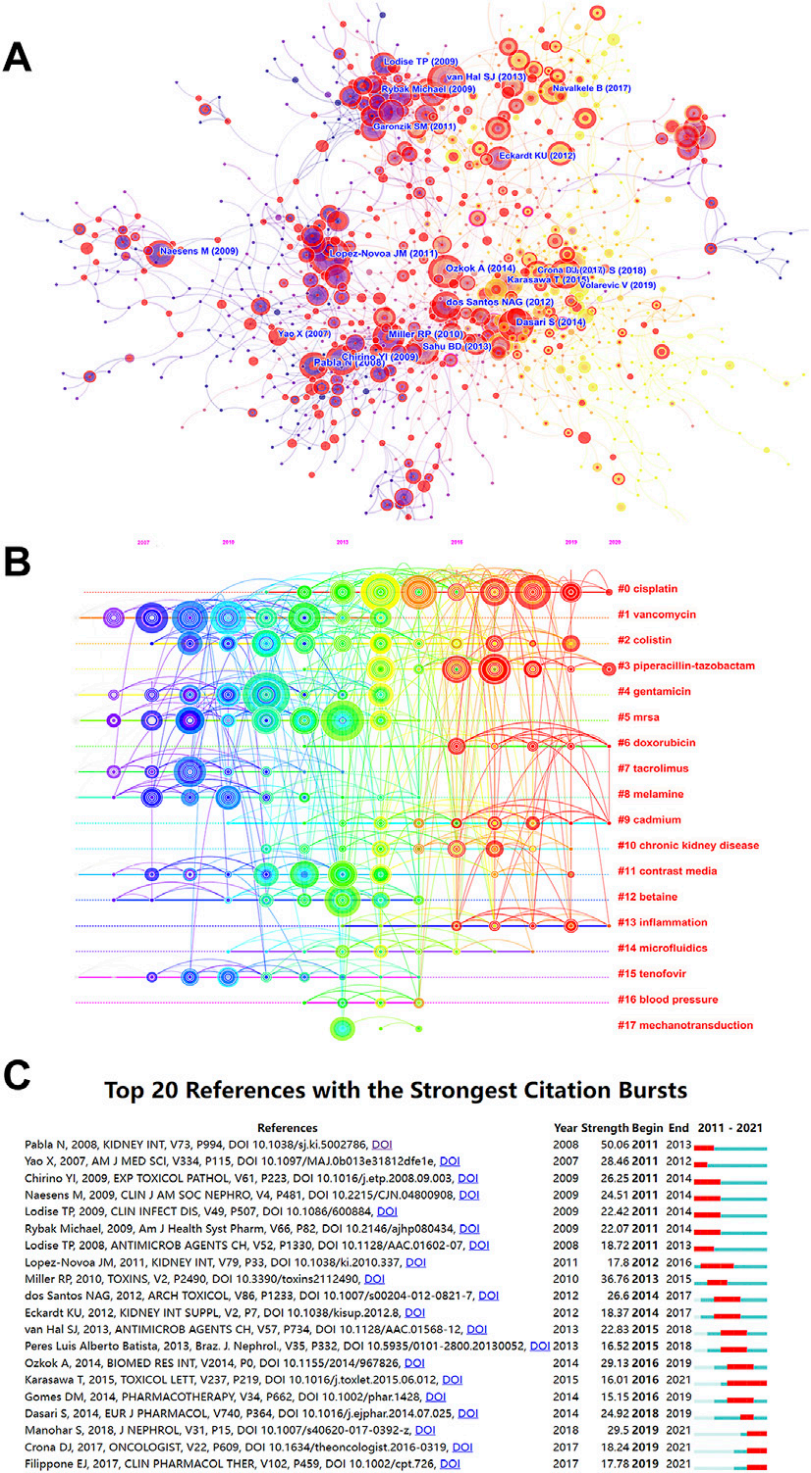
Analysis of most commonly cited references related to nephrotoxicity research. **(A)** Co-citation network of references. **(B)** Timeline view of reference cluster analysis. **(C)** Top 20 references with the strongest citation burst.

The top 10 highly cited literature on nephrotoxicity research were shown in Table 3. These studies mainly focus on antibiotic- and cisplatin-induced nephrotoxicity. Among them, the article published in *Nature* aroused wide concern, which demonstrated that the human nephrogenesis organoids model was representative of nephrotoxicity screening for future applications (Takasato et al., 2015). Another article proved that cisplatin-induced renal OS and apoptosis could be inhibited by exosomes from human umbilical cord mesenchymal stem cells (Zhou et al., 2013). To avoid the effect of publication year, the influence of time and co-citation were considered in citation analysis (Figure 7A). Interestingly, there were seven highly co-cited references related to cisplatin-induced nephrotoxicity (Table 4), which mainly focused on toxic mechanisms and protection strategies. These research studies (Pabla and Dong, 2008; Miller et al., 2010; Manohar and Leung, 2018) were most frequently co-cited with a higher centrality value, which demonstrated that the disorder of inflammation and cellular transporters could provoke renal tubular epithelial cells toxicity.

**TABLE 3:** Top 10 highly cited literature in nephrotoxicity research.

Rank	Citations	Author	Title	Source	If	Year	Doi
1st	928	Sellares, J	Understanding the Causes of Kidney Transplant Failure: The Dominant Role of Antibody-Mediated Rejection and Nonadherence	*American Journal of Transplantation*	8.086	2012	10.1111/j.1600–6143.2011.03840.x
2nd	669	Takasato, Minoru	Kidney organoids from human iPS cells contain multiple lineages and model human nephrogenesis	*Nature*	49.962	2015	10.1038/nature15695
3rd	514	Garonzik, S.M	Population Pharmacokinetics of Colistin Methanesulfonate and Formed Colistin in Critically Ill Patients from a Multicenter Study Provide Dosing Suggestions for Various Categories of Patients	*Antimicrobial Agents and Chemotherapy*	5.191	2011	10.1128/AAC.01733-10
4th	433	Jang, Kyung-Jin	Human kidney proximal tubule-on-a-chip for drug transport and nephrotoxicity assessment	*Integrative Biology*	2.192	2013	10.1039/c3ib40049b
5th	393	Zhou, ying	Exosomes released by human umbilical cord mesenchymal stem cells protect against cisplatin-induced renal oxidative stress and apoptosis *in vivo* and *in vitro*	*Stem Cell Research*	6.832	2013	10.1186/scrt194
6th	384	Marullo, Rossella	Cisplatin Induces a Mitochondrial-ROS Response That Contributes to Cytotoxicity Depending on Mitochondrial Redox Status and Bioenergetic Functions	*PLOS One*	3.240	2013	10.1371/journal.pone.0081162
7th	359	Wunderink, Richard G	Linezolid in Methicillin-Resistant *Staphylococcus aureus* Nosocomial Pneumonia: A Randomized, Controlled Study	*Clinical Infectious Diseases*	9.079	2012	10.1093/cid/cir895
8th	308	Kullar, Ravina	Impact of Vancomycin Exposure on Outcomes in Patients with Methicillin-Resistant *Staphylococcus aureus* Bacteremia: Support for Consensus Guidelines Suggested Targets	*Clinical Infectious Diseases*	9.079	2011	10.1093/cid/cir124
9th	295	Homan, Kimberly A	Bioprinting of 3D Convoluted Renal Proximal Tubules on Perfusable Chips	*Scientific Reports*	4.379	2016	10.1038/srep34845
10th	259	Kalghatgi, Sameer	Bactericidal Antibiotics Induce Mitochondrial Dysfunction and Oxidative Damage in Mammalian Cells	*Science Translational Medicine*	17.956	2013	10.1126/scitranslmed.3006055

**TABLE 4:** Top 10 co-citation references in nephrotoxicity research.

Rank	Frequency	Centrality	Author	Title	Source	IF (JCR 2020)	Year	Doi
1st	114	0.06	Pabla, N	Cisplatin nephrotoxicity: mechanisms and renoprotective strategies	*Kidney International*	10.612	2008	10.1038/sj.ki.5002786
2nd	99	0.08	Miller, Ronald P	Mechanisms of Cisplatin nephrotoxicity	*Toxins*	4.546	2010	10.3390/toxins2112490
3rd	88	0.02	Lopez-Novoa, Jose M	New insights into the mechanism of aminoglycoside nephrotoxicity: an integrative point of view	*Kidney International*	10.612	2011	10.1038/ki. 2010.337
4th	88	0.02	Ozkok, Abdullah	Pathophysiology of cisplatin-induced acute kidney injury	*Biomed Research International*	3.411	2014	10.1155/2014/967826
5th	84	0.05	van Hal, S. J.	Systematic Review and Meta-Analysis of Vancomycin-Induced Nephrotoxicity Associated with Dosing Schedules That Maintain Troughs between 15 and 20 Milligrams per Liter	*Antimicrobial Agents and Chemotherapy*	5.191	2013	10.1128/AAC.01568-12
6th	83	0.06	Manohar, Sandhya	Cisplatin nephrotoxicity: a review of the literature	*Journal of Nephrology*	3.902	2018	10.1007/s40620-017-0392-z
7th	81	0.01	Dasari, Shaloam	Cisplatin in cancer therapy: Molecular mechanisms of action	*European Journal of Pharmacology*	4.432	2014	10.1016/j.ejphar. 2014.07.025
8th	78	0.07	Guinaim dos Santos, Neife Aparecida	Cisplatin-induced nephrotoxicity and targets of nephroprotection: an update	*Archives of Toxicology*	5.153	2012	10.1007/s00204-012-0821–7
9th	76	0.05	Chirino, Yolanda I	Role of oxidative and nitrosative stress in cisplatin-induced nephrotoxicity	*Experimental and Toxicologic Pathology*	-	2009	10.1016/j.etp. 2008.09.003
10th	71	0.02	Naesens, Maarten	Calcineurin inhibitor nephrotoxicity	*Clinical Journal of the American Society of Nephrology*	8.237	2009	10.2215/CJN.04800908

### Cluster analysis of references

The high-frequency terms of nephrotoxicity were displayed by the timeline cluster analysis, including 18 categories (Figure 7B). In our study, the mean S of the 20 clusters was 0.9084, indicating that the clusters were convincing. Among them, “#0 cisplatin” was the largest cluster with 133 references, followed by “#1 vancomycin (105),” “#2 colistin (78),” “#3 piperacillin-tazobactam (75),” and “#4 gentamicin (74).” In the past 5 years, #0 cisplatin, #3 piperacillin-tazobactam, #9 cadmium, and #13 inflammation have dominated the core part of nephrotoxicity.

The top 20 references with the strongest strength citation burst represented the frontiers of the nephrotoxicity field, and the burst strength ranged from 15.15 to 50.06 while endurance strength ranged from 3 to 6 years by red squares (Figure 7C). The research focused on cisplatin-induced nephrotoxicity that maintained a long-term level of citation burst, which discussed the toxic mechanism of endoplasmic reticulum stress (ERS), inflammation, and DNA damage in cisplatin (Karasawa and Steyger, 2015). In the last 3 years, citation burst indicated that scientists mainly focused on the renoprotective mechanism and strategies of cisplatin (Crona et al., 2017; Manohar and Leung, 2018) and vancomycin-induced nephrotoxicity with the pharmacokinetics/pharmacodynamics metabolism (Filippone et al., 2017).

## Discussion

It is worth noting that nephrotoxicity constitutes an important factor restricting the development of drugs and clinical therapy, which contributes to AKI in 14.4% of patients and 19% of renal failure in critically ill patients (Uchino et al., 2005; Hoste et al., 2015). Therefore, the development of clinically applicable interventions for nephrotoxicity are particularly important for the prevention of kidney injury and the development of new drugs. Notably, a numerous number more than 20,000 nephrotoxicity articles have been published around the world, which could be tracked back to 1933 (Bell, 1933). To further predict the hotspots precisely and make suggestions for the future perspective, we finally utilized bibliometric technology to analyze the latest nephrotoxicity literature between 2011 and 2021 globally, providing a basic reference for scientists to discover the hotspots and frontiers of nephrotoxicity.

### General data

The output of studies is the basic indicator for reflecting the activity degree of nephrotoxicity; 9,342 publications were obtained based on WoSCC, and cisplatin, OS, antibiotics, and AKI were still the dominating player in this field. Our results indicated that the development of nephrotoxicity field showed a significant growth trend in nearly a decade, with the number of publications and citations in 2021 reaching 1,014 and 32,665, respectively. An increasing number of resources (funds, scholars) will be invested in this field, and nephrotoxicity research will gain more momentum.

As the main driving force, the United States and China are highly productive countries with frequent citations and higher H-index of nephrotoxicity literature. Interestingly, Asia had made significant contributions to nephrotoxicity research due to the productive and wide application of natural products, especially in traditional Chinese medicine components, including aristolochic acids, alkaloids, and flavonoids (Yang et al., 2018; Xu et al., 2020). In addition, the United States, England, and Germany were the core nodes, and the nephrotoxicity cooperation network was mainly concentrated in Europe and America, which particularly focused on pharmacology, pharmacy, urology, and nephrology.

However, the most prolific institutions were from Egypt and Saudi Arabia, including King Saud University and Cairo University. The institutions from the United States have closer cooperation with each other, which further strengthened its academic influence on nephrotoxicity research by maximizing its geographical advantages. Although Saudi Arabia ranked eighth in the number of publications, King Saud University ranked first in the number of publications and centrality in the cooperation network, which mainly focused on mechanism analysis of natural product protection against nephrotoxicity underlying OS and apoptosis, suggesting that it is quite professional in this field (Dkhil et al., 2014; El Gamal et al., 2014; Hagar and Al Malki, 2014). But in general, the lack of frequent global cooperation in institutions across different countries limits its development in nephrotoxicity research.

In the authors’ contribution, Kang, Ki Sung from South Korea was the productive author, the research studies mainly focused on the renoprotective of ginsenosides against cisplatin-induced nephrotoxicity through mitigating apoptosis and inflammation (Park et al., 2015; Han et al., 2016). Moreover, Li, Jian with the highest H-index had depth research in colistin-induced nephrotoxicity. Among them, the research of dosing guidance for intravenous colistin to reduce the risk of nephrotoxicity (Nation et al., 2017) and the research studies on the toxic mechanism of nephrotoxicity through ERS, mitochondrial, death receptor, and apoptosis were the highest cited articles (Dai et al., 2014; Park et al., 2015). Another contributor is Abdel-Daim, Mohamed M, who mainly focused on the renoprotective mechanism action of Spirulina platensis in deltamethrin-induced nephrotoxicity through inhibition of lipid peroxidation, NO, and OS (Abdel-Daim et al., 2013; Abdel-Daim et al., 2016). The previously mentioned authors have higher academic reputations in renoprotective research studies and have made a great contribution to the developments and advancements.

The dual-map analysis found that the fields of molecular, biology, and immunology were the major publications in nephrotoxicity research studies. In addition, the Journal Citation Reports (2020) (Khan et al., 2021) showed that most of the productive journals of nephrotoxicity were classified as Q1 or Q2. Among them, *Antimicrobial Agents and Chemotherapy* and *Food and Chemical Toxicology* were the most productive journals with higher H-index and IF than five concurrently, indicating that these journals were highly favored for scientists with convincing and mature outcomes to enhance their academic influence on nephrotoxicity.

### Knowledge base

The highly cited studies mainly focused on clinical dosing suggestions of antibiotics (Garonzik et al., 2011; Neely et al., 2014) and the renoprotective mechanisms of natural products research studies (Arjumand et al., 2011; Domitrovic et al., 2013). Notably, the co-cited network of references constitutes the knowledge base of the field. By combining the frequency and centrality of a reference, these references including mechanisms of nephrotoxicity and renoprotective in antibiotic, cisplatin, and calcineurin inhibitor that performed important knowledge-based functions were obtained. Here are the main findings.

Cisplatin, as a much sought chemotherapeutic agent, was still the dominant player of nephrotoxicity research, inducing apoptosis in tumor cells through DNA damage (Dasari and Tchounwou, 2014). At present, the major toxic mechanism in cisplatin-induced nephrotoxicity includes DNA damage, cytoplasmic organelle dysfunction, mitochondrial dysfunction, ERS, cell apoptosis, OS, and inflammation (Manohar and Leung, 2018). The tubulo-interstitial compartment is the major damage part (Ozkok and Edelstein, 2014). Notably, concurrent use of nephrotoxic medications (such as nonsteroidal anti-inflammatory drugs and iodinated contrast) are also considered risk factors. Many renoprotective measures have been studied, which are mainly employed for hydration/diuresis and monitoring of renal function. In addition, nephroprotective agents mainly included amifostine, amino oxyacetic acid, peroxisome proliferators-activated receptor agonists, and quercetin (dos Santos et al., 2012), which focused on inhibition of p53 targets, g-glutamyl transpeptidase and cysteine-S-conjugate b-lyase metabolism, cell death, OS, and inflammation (Pabla and Dong, 2008). However, it is unclear whether they reduce the cisplatin therapeutic effects in tumors concurrently.

OS is commonly caused by nephrotoxicity. Maintenance of mitochondria homeostasis plays a key role against OS to attenuate renal injury. Studies showed that OS could be activated with ROS accumulation to cause AKI by gap junction protein connexin 32 expression underlying mitochondrial apoptosis (Luo et al., 2015; Chen et al., 2019). In addition, antioxidant therapy had aroused a growing concern about nephrotoxicity. Synthetic and natural antioxidants defended oxidative injury by activating antioxidant enzymes, inhibiting NOSs, oxidases, and decomposing peroxide (Hassan et al., 2017; Forman and Zhang, 2021; Gao et al., 2021). SOD1 is a major antioxidant enzyme against OS through inhibition of ROS production, restoration of mitochondrial function, and attenuation apoptosis (Wang et al., 2021). Combining with targeting gap junction proteins and antioxidant strategies could be a novel perspective in mitochondrial damage of nephrotoxicity research.

As for antibiotics, the nephrotoxicity mechanism of aminoglycoside presents a novel insight, which concentrated on an integrative analysis of tubular obstruction and malfunction, further activated tubuloglomerular feedback, and caused renal vasoconstriction and mesangial contraction in gentamicin-induced nephrotoxicity (Lopez-Novoa et al., 2011). Similarly, concentration and duration of therapy contributed to a key factor in nephrotoxicity exposure. A study showed that patients with vancomycin troughs in excess of 15 mg/L were preferred to generate toxicity (van Hal et al., 2013). In summary, the research studies of nephrotoxicity mechanisms in cisplatin and different antibiotics constitutes the knowledge base of the nephrotoxicity field in the last decade, which has achieved great progress.

### Research hotspots

The integrative analysis and cluster of high-frequency keywords and references can identify hotspots and frontiers in the nephrotoxicity research field. Among them, research on cisplatin, piperacillin-tazobactam, doxorubicin, and cadmium was the hotspot in the nephrotoxicity field based on timeline cluster.

The frontiers research studies demonstrated that cisplatin-induced nephrotoxicity could be inhibited by preventing mitochondrial fragmentation and subsequent cell injury and death through sirtuin 3-dependent mitochondrial dynamics remodeling underlying honokiol intervention (Mao et al., 2022). Also, the role of the renoprotective effect of leonurine hydrochloride was achieved by inhibition of lipid peroxide-mediated ferroptosis and activation of Nrf2 (Hu et al., 2022). In addition, renalase agonists may be a new tool to enhance the renoprotective effects (Curry and McCormick, 2022). Studies have reported that a combination of vancomycin and piperacillin-tazobactam could induce additive nephrotoxicity through routine creatinine evaluation, but it does not worsen the level of kidney injury molecular-1 biomarker, suggesting that kidney function should be considered by integrative evaluation (Chang et al., 2022). Doxorubicin nephrotoxicity could be mitigated by nano-resveratrol through modulation of Beclin-1 and mammalian TOR (Alhusaini et al., 2022). It could also be protected by pirfenidone and vitamin D through inhibition of c-Jun N-terminal kinase-1 and monocyte chemoattractant protein-1 pathways (Hazem et al., 2022). Cadmium nephrotoxicity was induced by bromodomain-containing protein 4, further mediating lysosomal dysfunction, autophagy blockade, and OS (Gong et al., 2022). Biological oxidation (mainly glucuronidation) and apoptosis were the major stress responses in the cadmium treatment, especially in diabetes (Feng et al., 2022).

By analyzing burst citations of references, we hypothesized that the renoprotective mechanism and strategies of cisplatin-induced nephrotoxicity and antibiotic-induced nephrotoxicity will continuously become an academic trend in nephrotoxicity research (Karasawa and Steyger, 2015; Manohar and Leung, 2018). In addition, the latest research also focused on the subject of cisplatin hydration, suggesting that short-duration, low-volume with magnesium supplementation, mannitol forced diuresis, and oral post-hydration were essential for nephrotoxicity prevention (Crona et al., 2017). In vancomycin-induced nephrotoxicity with attributed rates of >10%, guideline-based trough levels of 15–20 mg/L have greater nephrotoxicity than levels <15 mg/L, and combination with piperacillin-tazobactam should be avoided. It is safe to use vancomycin in patients with exposure risks for AKI with therapeutic drug monitoring and antibiotic stewardship. But the cessation of vancomycin should be considered once patients develop AKI (Filippone et al., 2017).

New biomaterials, such as nanoparticles, dendrimers, micelles, liposomes, and nanogels, including nanomedicine, , which could offer more precise pharmacotherapy options with improved safety profiles, especially in cisplatin and antibiotics, could attenuate nephrotoxicity (Cooper et al., 2014; Desai et al., 2022). Reports showed that cisplatin conjugated to a polyphosphazene, lipid-coated cisplatin nanoparticle, and cholesterol-tethered platinum II-based nanoparticle could increase the antitumor effect and reduce nephrotoxicity (Sengupta et al., 2012; Guo et al., 2013; Patil et al., 2020). Similarly, reduced nephrotoxicity was found in paclitaxel, amphotericin B, and cyclosporine-based nanoparticle application (Wang et al., 2011; Guada et al., 2016; Liu et al., 2020). Collectively, the renoprotective mechanisms and new biomaterials applications in cisplatin and antibiotics constitute the hotspots and frontiers in the future of this field.

### Future perspective and suggestions

Combined with the knowledge base and hotspots, there are several suggestions worth noting for future perspectives against nephrotoxicity. 1) Identifying early new warning biomarkers is a critical research priority for nephrotoxicity prevention using multiomics approaches; for example, mass spectrometry imaging can give an insight into biomarkers on a spatial level, which contributes to biomarker localization and pathological processes combination. 2) Antioxidant therapy strategies should be utilized in further research studies against oxidative injury, which could protect mitochondrial damage to restore disorders of the kidney;. 3) New biomaterials with new technologies, including nanomedicine, are emerging as a new clinical favorite to reduce nephrotoxicity, which could achieve precise guidance for targeted drugs.

## Limitations

However, our research also has some limitations. 1) Although the most commonly used databases in scientometrics are WoSCC, Scopus, and PubMed, databases would be further needed in a comprehensive analysis, exhibiting extreme difficulty in analyzing multiple databases using available bibliometric software. 2) Only English articles were included, and nephrotoxicity related research in 2022 were not included; therefore, we may have missed some research hotspots.

## Conclusion

Although the quantity of publications in nephrotoxicity has been growing at a rapid pace, especially in the past decade, nephrotoxicity crucially limits drug development and clinical applications. Our results demonstrated that the recognition of nephrotoxicity has improved significantly as follows. 1) The United States and China are the leading contributors. 2) Main research foci are the mechanism of nephrotoxicity and renoprotective in cisplatin and antibiotics. 3) Future research studies will focus on cisplatin, piperacillin-tazobactam, vancomycin, and cadmium. However, exploring more sensitive diagnostic markers of nephrotoxicity, focusing on antioxidant therapy, and strengthening the management and application of natural product nephrotoxicity are urgently needed globally. Collectively, this study systematically analyzed the literature on nephrotoxicity and reported the research results of the last decade in multiple dimensions, which could lay the foundation for its in-depth development and expand its clinical application globally.

## Data Availability

The original contributions presented in the study are included in the article/Supplementary Material; further inquiries can be directed to the corresponding authors.
